# Microvascular dysfunction and aberrant network activity drive reduced brain oxygenation in a mouse tauopathy model

**DOI:** 10.21203/rs.3.rs-8205391/v1

**Published:** 2025-12-22

**Authors:** Sung Ji Ahn, Antoine Anfray, Yun Losson, Mirna El Khatib, Liping Qian, Belem Yoval-Sánchez, Gang Wang, Ping Zhou, Alexander Galkin, Laibaik Park, Sergei Vinogradov, Josef Anrather, Costantino Iadecola

**Affiliations:** 1 Feil Family Brain and Mind Research Institute, Weill Cornell Medicine New York, NY 10021, USA; 2 University of Pennsylvania, Department of Biochemistry and Biophysics, Perelman School of Medicine, and Department of Chemistry, School of Arts and Sciences, University of Pennsylvania, Philadelphia, PA 19104

**Keywords:** neurovascular coupling, nNOS, excitation-inhibition imbalance, O_2_ phosphorescence lifetime imaging, capillary stalling, capillary transit time heterogeneity

## Abstract

Tauopathies such as Alzheimer’s disease and frontotemporal dementia are leading causes of cognitive impairment, characterized by accumulation of hyperphosphorylated tau, a microtubule-associated protein, in brain. In addition to driving neural network hyperactivity and neuronal damage, tau disrupts neurovascular function, which may further contribute to disease pathogenesis. Using a mouse tauopathy model, we demonstrate that tau causes a profound breakdown of the normally well-coordinated segmental vasodilation induced by neural activity, resulting in dampened and delayed blood-flow increases, heterogeneous capillary perfusion, and frequent capillary stalling. These neurovascular alterations arise in the context of pronounced network hyperactivity and hypersynchrony, which combined with impaired neurovascular coupling, lead to reduced oxygen availability, episodic hypoxia, and disturbed metabolic homeostasis. Collectively, these findings identify reduced brain oxygenation driven by an imbalance between neuronal hyperactivity and diminished oxygen delivery as a previously-unappreciated early pathogenic consequence of tau accumulation and bolster the rationale for therapies to restore cerebral oxygenation.

## Introduction

Cerebrovascular alterations have emerged as early manifestations of neurodegenerative diseases associated with cognitive deficits, implicating neurovascular dysfunction in disease pathogenesis^1–4^. Patients with Alzheimer’s disease (AD) and frontotemporal dementia exhibit pronounced neurovascular alterations early in the disease course^4–7^, suggesting that pathogenic drivers of neurodegeneration, such as amyloid-β and the microtubule-associated protein tau, may exert their deleterious effects also by impairing vascular function. Indeed, amyloid-β has long been recognized to disrupt neurovascular regulation^8–10^, and growing evidence indicates that pathogenic forms of tau (p-tau) similarly induce neurovascular dysfunction^11–14^. As in patients, mouse models of tauopathies have revealed substantial disturbances in key homeostatic mechanisms regulating the neurovasculature. In particular, studies in 3-month-old PS19 and Tg4510 mice have demonstrated suppression of the cerebral blood flow (CBF) response to neural activity (functional hyperemia) preceding neurofibrillary tangles, neuroinflammation, vascular rarefaction, neurodegeneration, and cognitive decline^11^. This attenuation of functional hyperemia has been linked to the mis-localization of p-tau to dendritic compartments, where it interacts with the NMDA receptor adaptor protein PSD95^11^. Disruption of the PSD95–neuronal nitric oxide synthase (nNOS) complex reduces activity-dependent nitric oxide (NO) production^11^, a key mediator of neurovascular coupling in animals as in humans^15–20^. However, the effects of p-tau on the microvascular dynamics that orchestrate functional hyperemia and the resulting consequences for O_2_ delivery to the brain tissue have not been elucidated.

Functional hyperemia, also known as neurovascular coupling, is a fundamental property of the cerebral microvasculature that aligns the delivery of nutrients with the dynamic metabolic demands imposed by neural activity^21^. The underlying microvascular mechanisms involve a coordinated sequence of diameter changes across vascular segments that not only augment CBF but also enhance the homogeneity of red blood cell (RBC) flux through capillaries (transit time homogeneity), thereby optimizing O_2_ delivery to brain tissue^22–27^. Functional hyperemia increases local O_2_ availability beyond the amount consumed by neural tissue^28^, an O_2_ excess that leads to a decrease in deoxyhemogobin which underlies the blood-oxygen-level–dependent (BOLD) signal used in functional brain imaging (fMRI)^29–31^. Although the significance of this transient hyperoxia remains not completely understood^32^, it may serve to enhance the intravascular-to-tissue pO_2_ gradient to sustain oxygenation of cells located farther away from arterioles^33–36^. Despite this apparent O_2_ excess, spatiotemporal fluctuations in tissue pO_2_ have been detected in the healthy mouse brain, giving rise to small, transient hypoxic microdomains (pO_2_ < 18 mmHg)^37–39^. The frequency, size, and duration of these hypoxic “pockets” increase under conditions associated with neurovascular dysfunction or plugging of capillaries by blood cells (capillary stalling)^37–39^. The relationship between neural activity and CBF is particularly relevant to tauopathies, since p-tau disrupts the excitation–inhibition balance^40–43^ leading to network hyperexcitability and elevated metabolic demands^44, 45^. Thus, the finding that p-tau suppresses neurovascular coupling in the presence of neuronal hyperactivity raises the possibility that the resulting mismatch between O_2_ delivery and utilization impairs brain oxygenation. Such an energetic imbalance could contribute to the deleterious effects of p-tau on the brain.

To address this question, we used PS19 mice in the awake state and before the development of florid pathology to examine the impact of p-tau on the microvascular dynamics underlying functional hyperemia and their relationship to network activity and brain oxygenation. We found that p-tau profoundly disrupted the coordinated microvascular responses evoked by neural activity, resulting in delayed and attenuated CBF increases, capillary stalling, and heterogeneity of capillary flow. This disruption weakened the close correspondence between neuronal activity and the hemodynamic response, leading to impaired neurovascular coupling and reduced local perfusion despite neuronal network hyperactivity. Consequently, tissue oxygenation was decreased at rest and further reduced during neural activation, producing hypoxic transients. These alterations were accompanied by evidence of metabolic insufficiency, as revealed by NADH imaging. Collectively, our findings identify reduced brain O_2_ availability as a previously unrecognized early pathogenic consequence of p-tau accumulation that may exacerbate neuronal dysfunction and injury in tauopathies.

## Results

### 1. Segmental vascular responses to neural activity are dysregulated in PS19 mice

**Extended Data Figure 1:**
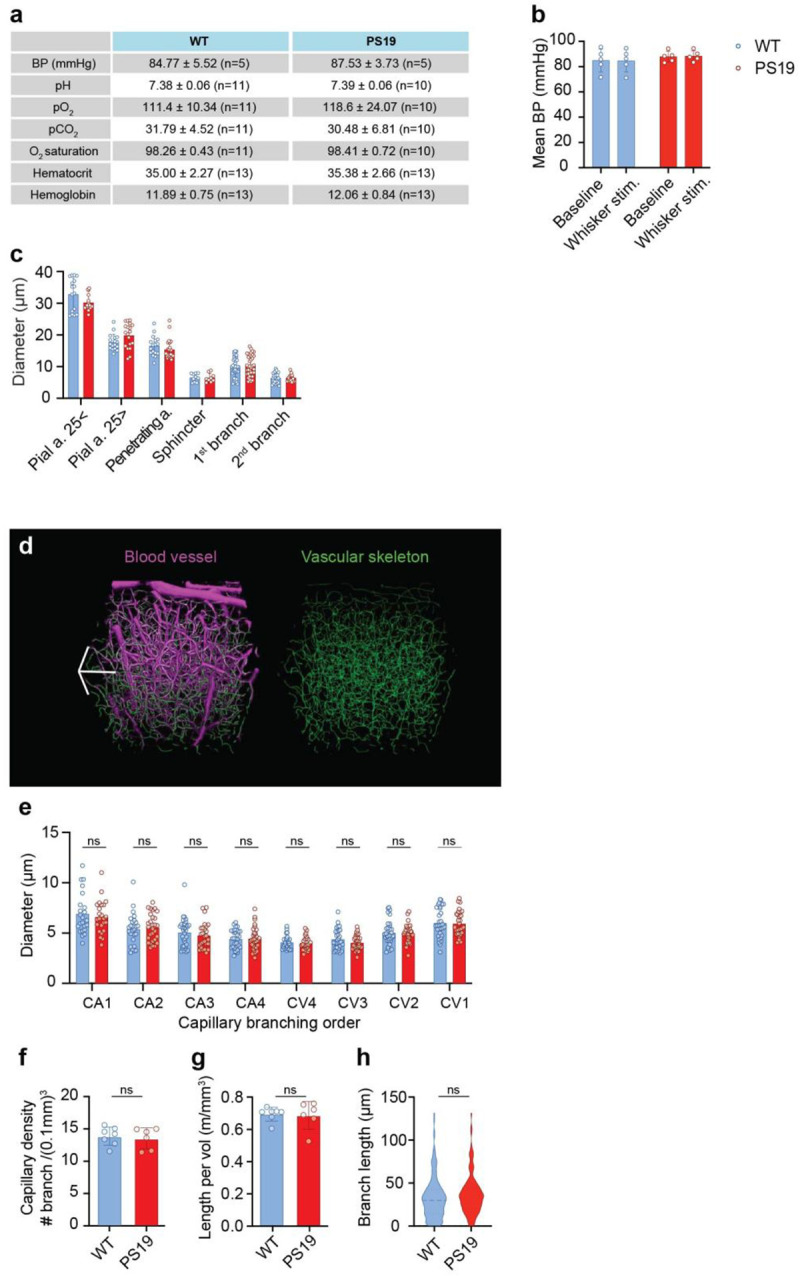
Physiological parameters, vessel diameter, and vascular network structure in WT and PS19 mice **a.** Blood pressure and arterial blood gases (pH, pO_2_, pCO_2_, O_2_ saturation), hematocrit, and hemoglobin in a representative set of awake mice during 2-photon imaging in the awake state. **b.** Blood pressure at baseline and during whisker stimulation was similar in WT and PS19 mice during 2-photon imaging in the awake state. Repeated measure Two-way ANOVA with Fisher’s LSD; n=5/group. **c.** Baseline diameters of vessels in Fig. 1e and f, from pial arteriole to the second downstream branch of the penetrating arteriole did not differ between WT (n=10) and PS19 mice (n=8). Two-way ANOVA and Tukey’s test. **d.** Representative 3D visualization of the vascular network, showing dextran-labeled blood vessels (magenta) and the corresponding vascular skeleton (green). 3D scale bar: 100 μm. **e.** Capillary diameters (1^st^ to 4^th^ branch order) did not differ between WT (n=7) and PS19 mice (n=6); two-way ANOVA and Tukey’s test. **f.** Capillary density, quantified as the number of capillary segments per unit brain volume, was similar between WT (blue; n=7) and PS19 mice (red; n=6); Welch’s t-test. **g.** Total capillary length per unit brain volume did not differ between WT (n=7) and PS19 mice (n=6); Mann-Whitney test. **h.** Length of capillary branches were comparable between WT (n=7) and PS19 mice (n=6); nested t-test.

**Figure 1:**
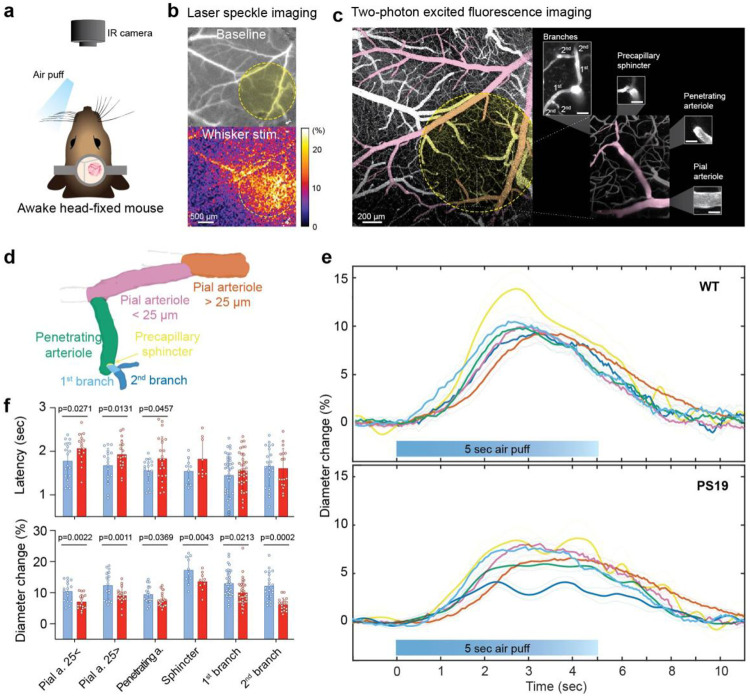
The segmental microvascular dilatation evoked by sensory stimulation is disrupted in PS19 mice. **a.** Experimental setup for awake, head-fixed mice with air puff stimulation. **b.** Laser speckle imaging was used to map the location of the whisker barrel cortex. **c.** Representative two-photon vascular map of the same region with the barrel cortex highlighted by the yellow circle in **b** (top). A penetrating arteriole supplying the barrel cortex and its downstream branches in the dash-lined white rectangle are magnified and labelled in the right panel. **d.** Reconstruction of the vascular segments shown in **e** and **f**. **e.** Mean percent diameter changes of individual vessel segments in WT (n=11) and PS19 mice (n=19) during stimulation (mean ± SE, see **d** for segment identification). **f.** Response latencies and peak diameter changes for individual vessel segments during stimulation in WT and PS19 mice (blue and red, respectively; animal numbers as in **e**). Data expressed as mean ± SD; two-way ANOVA with Tukey’s test (latency - vessel type: p=0.0171, genotype: p=0.0006, no interaction; % diameter change – vessel type: p<0.0001, genotype: p<0.0001, no interaction). P values from Tukey’s test are shown above bar graphs.

Male and female WT and PS19 mice (age 3 months), expressing the P301S tau mutation^11, 46^, were equipped with a cranial window and head fixation device, and habituated to the 2-photon imaging setup for awake imaging (Fig. 1a). On the day of the experiment, the whisker barrel cortex was localized by monitoring the increase in CBF induced by mechanical stimulation of the whisker (Fig. 1b). After i.v. injection of fluorescently labelled dextran for vascular visualization, mice were placed under the 2-photon microscope. In all imaging sessions, an infrared camera (IR) simultaneously captured whisker deflection and mouse behavior (Fig. 1a). No differences in blood pressure, both at rest or during air-puff stimulation, as well as blood gasses, which could confound the interpretation of the hemodynamic signals^47^, were observed between WT and PS19 mice (extended data Fig. 1a,b). Changes in diameter in connected microvascular segments were recorded in response to a 5-second air-puff stimulation of the whiskers (Fig. 1c,d). These vessels included pial arterioles (<25 and >25μm in diameter) penetrating arterioles, precapillary sphincters^48^, and first and second order capillary branches (<10μm) (Fig. 1c,d; see extended data Fig. 1c for baseline diameters). In WT mice, whisker stimulation elicited an orderly temporal sequence of segmental dilatation starting with the 1^st^ capillary branch, followed by sphincter, penetrating arteriole and pial arterioles, consistent with the previously described retrograde propagation of the vasodilation to neural activation^23–25, 49^ (Fig. 1e). In contrast, in PS19 mice the vasodilation was attenuated in all segments, and segmental vascular responses were significantly delayed, an effect reaching statistical significance in pial and penetrating arterioles (Fig. 1e,f). As a result, in PS19 mice the orderly sequence of segmental vasodilation was disrupted (Fig. 1e). Therefore, the microvascular adjustments underlying functional hyperemia are significantly altered in PS19 mice.

### 2. Reduced microvascular RBC fluxes and capillary transit time heterogeneity in PS19 mice

**Figure 2:**
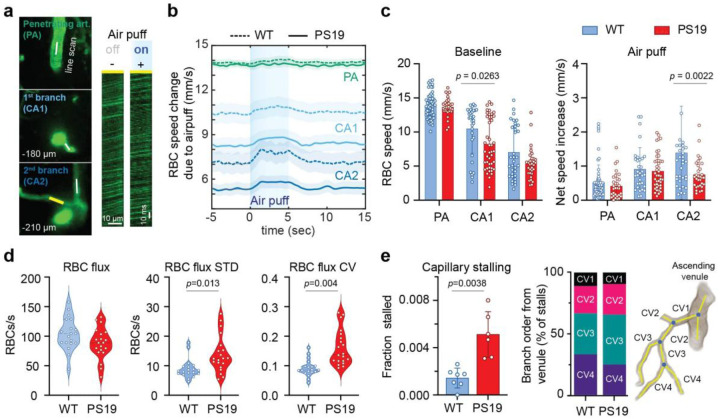
Impaired capillary perfusion dynamics in PS19 mice. **a.** Location of the line-scan measurements of red blood cell (RBC) speed in penetrating arterioles (PA) and downstream branches (CA1, CA2) during air-puff stimulation (left). Representative traces of line scans acquired at baseline (off) and during stimulation (on) in CA2 (marked by a yellow line in the left panel) (right). **b.** Traces of the RBC speed changes evoked by stimulation in PA, CA1 and CA2 (mean±SE). **c.** RBC speed at baseline and net speed increase during stimulation in PA, CA1 and CA2 of WT (dotted line; n=11) and PS19 mice (solid line; n=9). Mean±SD; two-way ANOVA with Tukey’s post hoc test for multiple comparisons. P values from Tukey’s post hoc test are shown above bar graphs. **d.** RBC flux and flux variability reflecting capillary transit time heterogeneity were quantified in capillary branches draining into ascending venules. STD: standard deviation; CV: coefficient of variance; n=5 per group, nested t-test. **e.** Stalled capillary segments assessed in branches draining into ascending venules in WT (n=7) and PS19 mice (n=6), labelled as shown in the cartoon on the right; mean±SD; Welch’s t-test.

Next, we investigated the impact of the attenuated and disorganized vasodilatatory response on microvascular perfusion, assessed by RBC line scanning (Fig. 2a)^11, 50^. No difference in capillary architecture (density, diameter, and branch length) was observed between WT and PS19 mice (extended data Fig.1d–f). In PS19 mice, baseline RBC speed was reduced mainly in the first capillary branches (Fig 2b), while the increase in RBC speed evoked by whisker stimulation was reduced in the second capillary branches (Fig. 2c). Since efficient capillary exchange depends on the homogeneity of capillary transit time^27^, we assessed RBC flux in the terminal capillaries draining into venules, as previously described^51^. In PS19 mice, capillary RBC flux tended to be lower and more heterogenous as assessed by the spread of the standard deviation and coefficient of variation of RBC fluxes^51^ (Fig. 2d).

Capillaries are not continuously perfused and segments with temporary interruption of RBC flux are observed, a phenomenon termed capillary stalling^52, 53^. Since reduced RBC flux and capillary flow heterogeneity may increase capillary stalling^52–55^, we investigated if stalled capillaries were increased in PS19 mice. We found that the fraction of capillaries with stalled flow, better appreciated in the pre-venular branches of the capillary network, was greater in PS19 than in WT mice (Fig. 2e). These observations, collectively, demonstrate a profound dysregulation of the microvascular responses to activation leading to heterogeneity of capillary RBC fluxes and increased capillary stalling, factors which may reduce the efficiency of capillary exchange.

### 3. Neural network hyperactivity and hypersynchrony in PS19 mice

**Figure 3.**
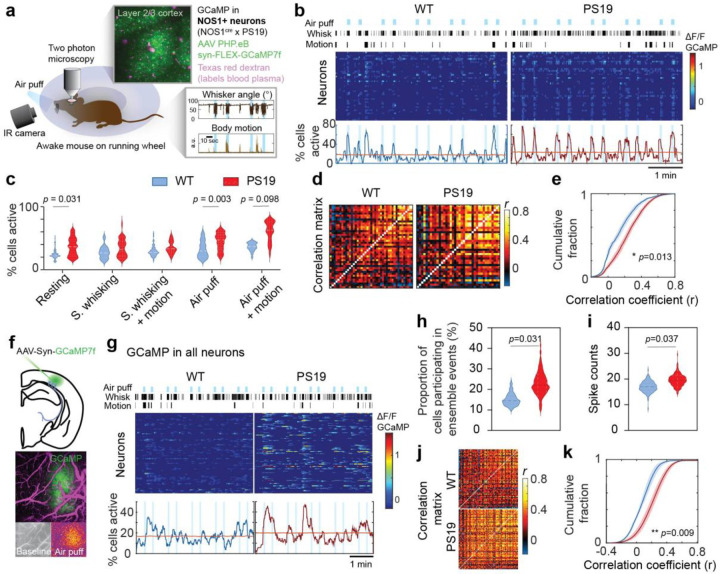
Increased neuronal activity and functional connectivity in PS19 mice. **a.** Cartoon illustrating the experimental setup. An awake mouse on a running wheel is shown receiving whisker air-puffs. An infrared (IR) camera tracks whisker angle and body motion (inset bottom right). Two-photon imaging of cortical layer 2/3 with GCaMP7f expression in NOS1^+^ neurons and intravascular dextran labeling of vessels (top inset). **b.** Representative neuronal population activity in WT and PS19 mice with GCaMP expression in nNOS neurons. Top markings show air-puffs (blue), whisking and motion detected by the infrared camera. The middle panel shows ΔF/F raster plot across neurons. Bottom panel shows corresponding neural ensemble traces, and significant ensemble activity threshold (red line) assessed as described in the methods. **c.** Quantification of the percentage of active cells exceeding the ensemble threshold across behavioral states in WT (2,546 data points from 6 recordings in 3 mice), and PS19 mice (3,027 data points from 7 recordings in 4 mice); data shown as median and interquartile range; nested t-test. **d.** Raster plots depicting changes in activity (ΔF/F) over time for a representative nNOS neuronal populations in WT and PS 19 mice. Each row represents a single cell. **e.** Cumulative distributions of pairwise correlation coefficients (r), showing a rightward shift in PS19 mice (n=8,165 correlations from 7 recordings in 4 mice) compared to WT mice (n=3,611 correlations from 6 recordings in 3 mice); nested t-test. **f.** Injection of AAV-Syn-GCaMP7f in barrel cortex to express GCaMP7F in all neurons. The GCaMP7F expression overlaps with the barrel cortex as shown by laser speckle CBF monitoring (bottom). **g.** Raster plot of representative general neuronal population activity (AAV-Syn-GCaMP7f) in WT and PS19 mice. **h.** Proportion of cells participating in ensemble events in PS19 (2,393 data points from 6 recordings in 3 mice) vs. WT mice (2,652 data points from 6 recordings in 3 mice); data shown as median and interquartile range; nested t-test. **i.** Spike counts are higher in PS19 mice (n=913 from 6 recordings in 3 mice) than in WT mice (n=601 from 6 recordings in 3 mice). Data shown as median and interquartile range; nested t-test. **j.** Representative functional connectivity matrices showing increased neuronal connectivity in PS19 mice **k.** Cumulative distributions of pairwise correlation coefficients among neurons (r), showing a rightward shift in PS19 mice and stronger functional connectivity (n=143,384 correlations from 6 recordings in 3 mice) in WT mice n=68,870 correlations from 6 recordings in 3 mice); nested t-test.

To gain insight in the neurovascular correlates of the alterations in magnitude and segmental dynamics of the neurovascular response to activation, we investigated the relationship between neural activity and microvascular responses. We have previously shown that the attenuation in functional hyperemia in PS19 mice is due to p-tau dampening NO production by nNOS neurons^11^. Based on these previous observations, we investigated the relationship between nNOS neuron activity and microvascular responses. To this end, we administered systemically AAVs expressing GGaMP7f (PHP.eB-syn-FLEX-GGaMP7f) in PS19 × NOS1^Cre^ crosses, which resulted in GGaMP7f expression in nNOS neurons (see ref.^47^ for details). NOS1^Cre^ mice treated with the AAV served as controls. Mice were equipped with a cranial window, habituated as described above and placed under the 2-photon microscope with video monitoring of whisker deflection and behavior (Fig. 3a). Despite being head-restrained, the mice were able to adjust their body posture and occasionally engaged in grooming behavior while on the running wheel. First, we examined patterns of Ca^2+^ activity of nNOS neurons in layer 2/3 by quantifying neuronal ensemble activity as the proportion of cells active at the same time over the recording period^56^. Mouse behavior was classified into five defined states: rest (no motion or whisking), spontaneous whisking, spontaneous whisking with motion (e.g., grooming or upper body postural changes during ambulation), air-puff–evoked whisking, and air-puff–evoked whisking with motion (Fig. 3b,c). We compared the nNOS neuron ensemble activity corresponding to each behavior and found that ensemble activity was significantly elevated at rest and during air-puff plus motion in PS19 mice compared to WT mice (Fig. 3c), with other behaviors showing a trend toward increased ensemble activity (Fig. 3c). To assess the functional connectivity among nNOS neurons, we constructed a correlation matrix of Ca^2+^ activity across all cells within a given field of view^47, 57^ (Fig. 3d). In PS19 mice, a greater proportion of nNOS neurons exhibited elevated synchronous activity (Fig. 3d,e).

We then examined whether hypersynchronous activity was also present across the general neuronal population. To this end, we injected AAVs expressing GGaMP7f (AAV9-syn-GGaMP7f) directly into the cortex in WT and PS19 mice, which resulted in widespread neuronal GGaMP7f expression (Fig 3f). We applied the same sensory stimulation protocol used for nNOS neurons, and imaged spontaneous and evoked Ca^2+^ transients in neurons of layer 2/3 of the somatosensory cortex. Unlike in nNOS neurons, ensemble activity in the general neuronal population did not show a tight correlation with air-puff stimulation (Fig. 3g), consistent with previous reports^58–60^. However, PS19 mice exhibited a higher proportion of hyperactive neurons (Fig. 3g–i) and increased synchrony (Fig. 3j,k). To provide additional evidence of neuronal hyperactivity we used patch clamping in brain slices of WT and PS19 mice. Consistent with the Ca^2+^ imaging data, we found that neurons tended to be hyperactive, as shown by increased resting potential and increased firing rate to a depolarizing pulse (extended data. Fig. 2a–d). These results, collectively, show that in PS19 mice nNOS neurons and the general neuronal population display increased functional connectivity and hypersynchronous network activity.

### 4. Reduced neurovascular coupling efficiency in PS19 mice

**Figure 4:**
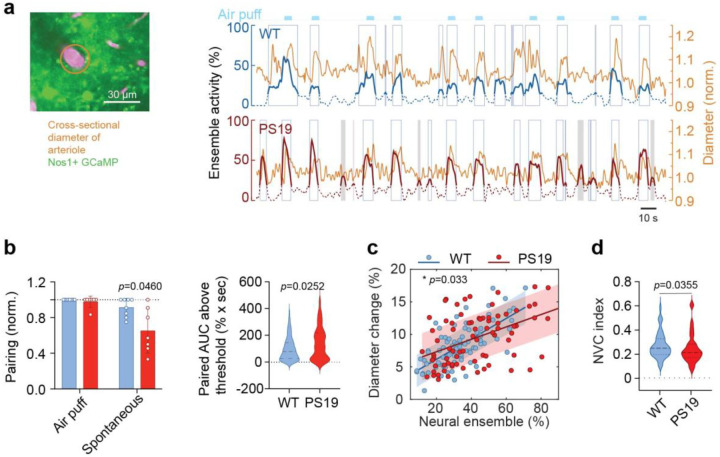
Attenuated vasodilatation evoked by neural activity in PS19 mice **a.** Two-photon image of NOS1 neuron Ca^2+^ activity (green) and a penetrating arteriole labeled with intravascular dextran (magenta) (left). Representative traces of ensemble activity (left axis) and normalized arteriole diameter (right axis) over time; dotted lines denote non-statistically significant ensemble activity and solid lines statistically significant activity (see Methods). Open rectangles denote ensemble activity associated with arteriolar vasodilatation and shaded rectangles denote activity not paired to vasodilatation. **b.** Ensemble events paired with vasodilatation. Pairing was defined as the number of ensemble events associated with vasodilatation divided by the sum of all events. Air puff stimulation was always associated with vasodilatation, although the magnitude of the response was attenuated in PS19 mice compared to WT mice (Fig. 1e). In PS19 mice, spontaneous ensemble activity was not reliably associated with vasodilatation. Each data point was obtained from 7 5-min recordings in 4 PS19 mice or 8 recordings in WT mice; mean ± SD; Mann-Whitney test. The violin plots (right) indicate that stronger ensemble activity was required to evoke paired vascular responses in PS19 mice. WT: n=127 ensemble events; PS19: n=96 ensemble events. Data shown as median and interquartile range; Mann-Whitney test. **c.** Relationship between neural ensemble activity and arteriole dilation. Scatter plots show percent diameter change as a function of ensemble activity for WT and PS19 mice. Linear regression lines and shaded 95% confidence intervals are shown. Regression lines were compared using ANCOVA (diameter % change; ensemble × genotype); p-value shown on plot. **d.** Neurovascular coupling index (NVC)^61^ is reduced in PS19 mice. Mann-Whitney test. WT: n=85 from 3 mice, PS19: n=77 from 3 mice.

Since Ca^2+^ activity in nNOS neurons contributes to functional hyperemia by producing NO^47^, a response attenuated in PS19 mice^11^, we next examined the relationship between nNOS Ca^2+^ activity and dilation of local penetrating arterioles. In WT mice arteriolar dilatations consistently followed spontaneous or air puff-induced neuronal ensemble activity (Fig. 4a). However, in PS19 mice, spontaneous ensemble activity did not always induce vascular responses (Fig. 4a). Only the stronger activity induced by whisker puffs was consistently paired with vasodilation (Fig. 4b), albeit attenuated compared to WT mice (Fig. 1e). Quantification of ensemble activity revealed that a greater nNOS neuronal ensemble activity was required to evoke vasodilation in PS19 mice compared to WT mice (Fig. 4b), suggesting reduced coupling between nNOS activity and the vascular response. To further investigate this, we correlated the magnitude of nNOS ensemble activity with the corresponding arteriolar dilation (Fig. 4c). We found that in PS19 mice the scatter of the data was increased, and the slope of the regression line was reduced, indicating that a given level of ensemble activity evoked a smaller vasodilation than in WT mice (Fig. 4c). To further quantify this effect, we calculated a neurovascular coupling index defined as the ratio of arteriolar diameter increase to neural ensemble activity^61^. PS19 mice exhibited a significantly lower neurovascular coupling index, indicating reduced coupling between neural activity and vascular response (Fig. 4d). Together, these findings suggest that neurovascular coupling is inefficient in PS19 mice, as expected from the reduction in NO production by nNOS neurons^11^. Accordingly, a higher level of nNOS neuron Ca^2+^ activity is required to elicit a vascular response, and even when this activity is sufficient, the resulting vasodilation is attenuated compared to WT mice.

### 5. Reduced intravascular pO2 and increased O2 extraction fraction in PS19 mice

**Figure 5.**
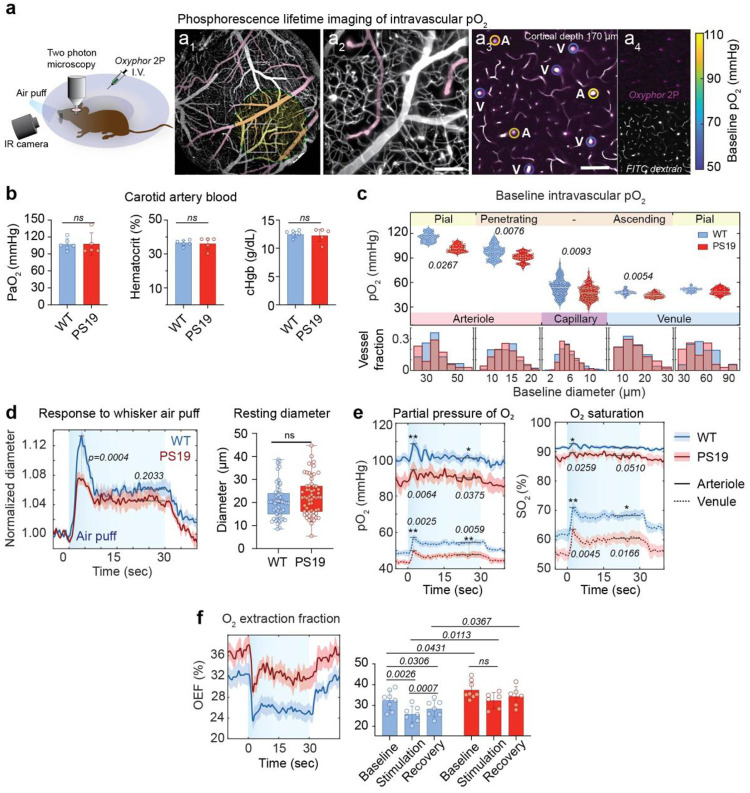
Reduced intravascular pO2 in microvessels of PS19 mice **a.** Cartoon illustrating the experimental setup. Oxyphor 2P was injected i.v. for intravascular pO_2_ measurement. **a**_**1**_: Pial surface with the barrel cortex highlighted in yellow. **a**_**2**_: Zoomed view of the white square in **a**_**1**_ showing the surface microvasculature at higher magnification; **a**_**3**_ pO_2_ in arterioles (A) and venules (V) in layer 2/3 170 μm below the surface. The circle surrounding the vessels is color coded to indicate pO_2_ values (calibration scale on the right). All scale bars are 100 μm. **b.** Carotid artery pO_2_ (assessed by blood-gas analysis), hematocrit, and hemoglobin, showing no difference between WT (n=6) and PS19 mice (n=5). **c.** Baseline pO_2_ measurements in arterioles capillaries and venules in layer 2/3 of the barrel cortex. Corresponding vessel diameter distributions are shown below; median and interquartile range; n=8/group; nested t-test. **d.** Normalized 30 seconds whisker stimulation evoked dilation of pial arterioles in WT and PS19 mice (n=6/group). Statistical comparisons were performed at the first and second peaks. A mixed effects model was used (main effects of genotype: p=0.0008; peaks: p <0.0001; interaction p=0.0484) (see Methods). Fisher’s LSD p-values are indicated on the plot. Resting diameters of the corresponding vessels are shown in the right panel. Data shown as median and interquartile range; Welch’s t-test on means; all vessels in 6 mice/group are plotted. **e.** pO_2_ (left) and O_2_ saturation (sO_2_; right) responses in arterioles and venules during whisker stimulation in WT (n=7) and PS19 mice (n=6). A mixed effects model was used (main effect genotype: arteriole pO_2_ p=0.215, venule pO_2_ p=0.0065, arteriole SO_2_ p=0.0443, venule SO_2_ p=0.0101; Fisher’s LSD p-values are indicated on the plot). **f.** Mean O_2_ extraction fraction (OEF) (left) and quantification at baseline, during stimulation and recovery in WT (n=7) and PS19 mice (n=6)(right). Mean ± SE; a mixed effects model was used (genotype: p=0.0241; time: p=0.0007; Tuckey’s test p values are indicated on the plot).

Considering the lack of the expected rise in CBF in response to the increased neural activity, and the reduced efficiency of microvascular O_2_ exchange associated with capillary transit time heterogeneity and stalling, we wondered if the delivery of O_2_ to the brain was reduced in PS19 mice. To examine this possibility, we assessed intravascular oxygenation using O_2_ phosphorescence lifetime imaging with the O_2_ probe Oxyphor 2P^62, 63^ (Fig. 5a). First, we confirmed that pO_2_, hemoglobin, and hematocrit levels in the carotid artery blood supplying the brain did not differ between WT and PS19 mice (Fig. 5b). Then, we measured intravascular pO_2_ in different segments of the somatosensory cortex microvasculature after intravenous administration of Oxyphor 2P (Fig 5a). In PS19 mice, baseline pO_2_ was significantly reduced in pial arterioles (-12.3 ± 3.6%), intracerebral vessels (penetrating arterioles: −12.3 ± 3.9 %; capillaries: −6.7 ± 1.9 %; ascending venules: −5.0 ± 1.5%), but not in pial venules (p>0.05), compared to WT mice (Fig. 5c). To evaluate stimulus-evoked intravascular O_2_ dynamics, we used a 30 second-long air-puff whisker stimulus to better resolve the temporal profile of the intravascular pO_2_ changes induced by activation. The 30-second stimulus elicited a biphasic pial artery dilation, with an initial peak at 2–3 seconds followed by a subsequent shallow peak at 20–25 seconds, both of which were attenuated in PS19 mice (Fig. 5d). In WT mice, whisker air-puff stimulation evoked increases in pO_2_ that were largest at stimulus onset and more sustained and pronounced in venules than in arterioles.^35^. In PS19 mice, baseline intravascular pO_2_ levels were reduced (Fig. 5e), as were the calculated O_2_ saturation (SO_2_) values^51, 64, 65^ (Fig. 5e). Based on the difference between arteriolar and venular SO_2_, we calculated the fraction of O_2_ extracted and metabolized by the brain (O_2_ extraction fraction, OEF)^33, 34, 51^. In PS19 mice, the baseline OEF was significantly elevated, suggesting that, unlike in WT mice, the heightened O_2_ demands were not met by an adequate delivery of CBF resulting in a higher OEF (Fig. 5f). In WT mice, neural activation by the air puffs induced the well-established decrease in OEF (Fig. 5f)^34^. The reduction in OEF is thought to result from the larger than needed CBF increase induced by functional hyperemia, leading to reduced capillary transit time and less time available for O_2_ exchange^33^. However, in PS19 mice the activation-induced drop in OEF was blunted and did not reach significant levels, suggesting a more precarious oxygenation of the brain tissue (Fig. 5f). These observations, collectively, indicate that although the pO_2_ of the blood supplying the brain through the carotid artery does not differ between WT and PS19 mice, the baseline pO_2_ in cerebral arterioles, capillaries and venules is lower in PS19 mice, and associated with a higher OEF. With activation, the OEF reduction is dampened in PS19 mice, reflecting the insufficient CBF increase needed to balance the increased O2 demands and attesting to the less efficient O_2_ delivery to the brain.

### 6. Reduced tissue oxygenation and O_2_ depletion during activation in PS19 mice

**Figure 6:**
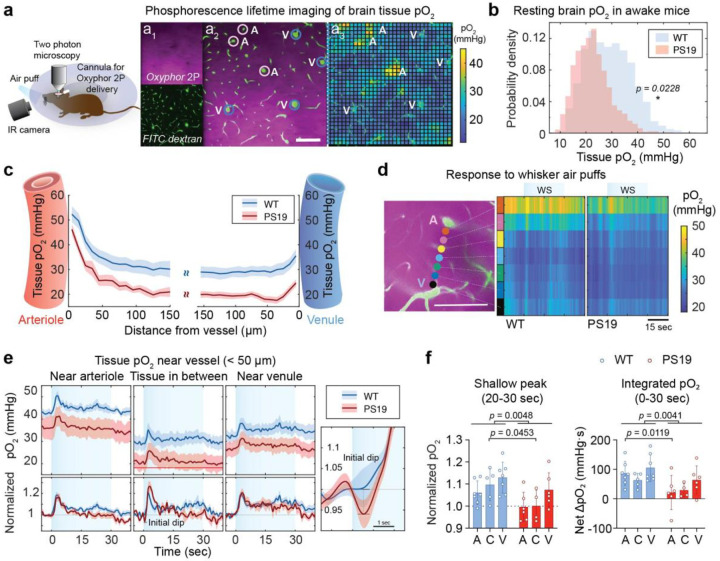
Brain tissue pO_2_ is reduced in PS19 mice reaching hypoxic levels during activation **a.** Cartoon illustrating the experimental setup for two-photon phosphorescence lifetime imaging of tissue pO2. Oxyphor 2P was injected into the brain parenchyma; **a**_**1**_: top: phosphorescence image of Oxyphor 2P injected in brain parenchyma; bottom: vasculature visualized by FITC dextran i.v. injection: **a**_**2**_: merged Oxyphor 2P and FITC dextran images with arterioles and venules labelled based on vasomotion and direction of flow; **a**_**3**_: calculated tissue pO2 values in brain tissue. pO2 is higher around arterioles than around venules. **b.** Distribution of resting tissue pO_2_, showing a leftward shift toward lower oxygen levels in PS19 mice; 1,792 measurements; 3 mice/group; nested t-test. **c.** Tissue pO_2_ plotted as a function of distance from the nearest vessel type, illustrating reduced tissue oxygenation across the microvascular network in PS19 mice. Mean ± SE; 10 arterioles, 16 venules from WT mice (n=4); 3,934 measurements. 10 arterioles, 14 venules from PS19 mice (n=3); 3,250 measurements; Two-way ANOVA (main effect vessel types: p=0.0013, genotype: p=0.0003). **d.** Color coded ROIs (dots) indicating the location of pO_2_ measurement between arteriole and venule (left). Heat map of tissue pO_2_ from near arterioles to venules measured at the color-coded ROI shown on the left panel. **e.** Changes in pO_2_ during stimulation near arterioles, venules, and the intervening capillary bed (mean ± SE; WT: n=7 (31 sites), PS19; n=6 (27 sites). The red line in the middle panel (tissue in between) marks the hypoxic threshold (pO2< 18 mmHg). The enlarged panel on the right shows the initial dip (mean ± SEM). **f.** Tissue pO_2_ at the shallow peak after 20–30 seconds stimulation. In WT mice, pO_2_ rises progressively from arteriole (A) capillaries (C) and venules (V), while in PS19 mice pO_2_ returns to baseline despite continuous stimulation. The right panel shows the net change in pO_2_ across the 30 seconds stimulation time, significantly lower in PS19 compared to WT; WT: n=7; PS19: n=6; Two-way ANOVA and Tukey’s test; p values are indicated on the plot. Scale bars in **a** and **d**: 100 μm.

To assess the impact of the reduced intravascular pO_2_ on brain oxygenation, we measured pO_2_ in the brain tissue. Oxyphor 2P was slowly delivered into the brain parenchyma through a chronically implanted cannula (Fig. 6a). Examination of the distribution of tissue pO_2_ in a 509×509μm^2^ area of layer 2/3 showed that the higher-range values were markedly diminished in PS19 compared to WT mice, leading to a shift of the O_2_ distribution histogram towards lower values (Fig. 6b). consequently, tissue pO_2_ in PS19 mice (22.1±2.7 mmHg) was on average 24% lower than in WT mice (29.3±3.0) (Fig. 6b). At the local level, the difference in tissue pO_2_ between WT and PS19 mice increased with the distance from arterioles (Fig. 6c,d). We then examined how tissue pO_2_ responded to brain activation by delivering a 30-second-long air-puff stimulus. To improve temporal resolution, we reduced spatial sampling by measuring tissue pO_2_ at discrete points between an arteriole and a venule (Fig. 6d). Tissue pO_2_ measurements were classified as located near arterioles (<50μm from arteriole), near venules (<50μm from venules), or >50μm from either vessels^38^ (Fig. 6e; Tissue in between). In WT mice, air puffs increased tissue pO_2_ with an initial peak at 2–4 seconds followed by a shallow peak at 20–30 seconds (Fig. 6e). In PS19 mice, baseline tissue pO_2_ was reduced near arterioles, venules, and in the tissue in between, and stimulation increased pO_2_, with an initial peak that did not differ from that observed in WT mice (Fig. 6e). However, in tissue farther form arterioles the pO_2_ peak was preceded by a prominent “dip”, not observed in WT mice, likely reflecting delayed O_2_ delivery to the tissue from upstream arterioles combined with the increased O_2_ demand of neuronal hyperactivity (Fig. 6e). As the simulation continued, tissue pO_2_ kept declining toward pre-stimulation levels, reflecting insufficient O_2_ delivery during activation (Fig. 6f). To determine whether tissue pO_2_ reached hypoxic levels we quantified the fraction of tissue pO_2_ measurements away from arterioles that fell below 18 mmHg^39, 66^. We observed that 14.2 % of tissue pO_2_ measurements (n=25) were in the hypoxic range, compared to only 1.33% in WT mice (n=30). Overall, tissue pO_2_ during activation was lower in PS19 compared to WT mice (Fig. 6f). These findings indicate that PS19 mice experience impaired O_2_ delivery and, consequently, tissue O_2_ depletion during activation, leading to reduced O_2_ availability and, potentially, more frequent episodes of transient hypoxia.

### 7. NADH imaging reveals altered metabolic adaptation to activation in PS19 Mice

**Figure 7:**
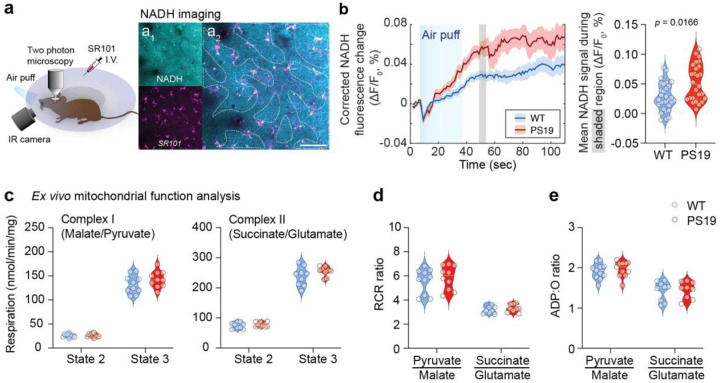
Post-stimulus NADH recovery is impaired in PS19 mice **a.** Cartoon illustrating the experimental setup for two-photon NADH imaging at a cortical depth of 65μm. **a**_**1**_: NADH autofluorescence (top), SR101 fluorescence (bottom); **a**_**2**_ merged image with outlined areas selected for imaging to exclude vessel shadows. Scale bar: 100 μm. **b.** NADH changes induced by whisker air puff stimulation (mean ± SE). The violin plots on the right show mean NADH values at the time window indicated by the shaded on the left panel; 30 measurements from WT (n=8), 26 measurements from PS19 mice (n=5). Data shown as median and interquartile range; nested t-test; p value indicated on the plot. **c.** Complex I – (pyruvate/malate, left) and complex II – supported (succinate/glutamate, right) respiration of intact neocortical mitochondria, showing state 2 (substrate only) and state 3 (ADP–stimulated) respiration. **d.** Respiratory control ratios (RCR), reflecting mitochondrial integrity in WT and PS19 mice. **e.** Mitochondrial phosphorylation efficiency measured as ADP:O ratios were similar between WT and PS19 mice. Panels **c-e:** data shown as median and interquartile range; mixed-effects ANOVA; Fisher’s LSD; n=3/group.

To evaluate stimulus-evoked metabolic responses in the cortex, we performed two-photon imaging of NADH. In the brain, NADH autofluorescence changes have long been used to monitor shifts in oxidative phosphorylation^34, 67–70^. As a key electron carrier NADH is generated in the cytosol and mitochondria and oxidized to NAD^+^ during oxidative phosphorylation. Increased NADH fluorescence is thought to reflect elevated metabolic demands and has been used extensively as a marker of energy metabolism homeostasis^34, 68, 69, 71, 72^. Since NADH fluorescence (~450–460 nm) is highly susceptible to hemodynamic interference, we corrected the autofluorescence signal using SR101 as a nonfunctional fluorophore, as previously described^68^ (Fig. 7a). In WT mice, whisker air puffs elicited a biphasic response characterized by an initial dip in NADH signal reflecting increased oxidative metabolism, followed by the well-described signal overshoot^73, 74^. In PS19 mice, stimulation elicited a dip like that of WT mice, but the overshoot was much more pronounced (Fig.7b). Since the NADH overshoot is enhanced during mild hypoxia^68^, this exaggerated response in PS19 mice supports the conclusion that tissue O_2_ is reduced in this model.

Considering that NADH levels are related to mitochondrial metabolism^72, 75, 76^, we examined selected indices of mitochondrial function in isolated brain mitochondria of WT and PS19 mice^77, 78^. We measured O_2_ consumption rates through complex I (malate/pyruvate) and complex II (succinate/glutamate) substrates under both non-phosphorylating (state 2) and ADP-stimulated (state 3) conditions (Fig. 7c,d). In addition, we evaluated the respiratory control ratio, reflecting mitochondrial integrity, the ADP:O ratio (oxidative phosphorylation efficacy), mitochondrial ROS production, and the membrane potential response to ADP load (capacity to maintain polarization during energy demand) (Fig. 7d,e; extended data Fig. 3a–c). Our analyses revealed no significant differences between WT and PS19 mice across all functional measures. To further assess whether the observed changes could stem from altered mitochondrial content rather than activity, we quantified the absolute levels of mitochondrial complex I and α-ketoglutarate dehydrogenase. These measurements also showed no differences between genotypes, reinforcing the conclusion that mitochondrial content and function are preserved in PS19 mice (extended data Fig. 3d). Taken together, these observations strongly indicate that reduced tissue O_2_ availability impairs metabolic adaptation to the increased energy demands of the active brain.

## Discussion

We investigated the microvascular mechanisms underlying the attenuation of functional hyperemia induced by p-tau and its implications for the O_2_ supply to the brain tissue. In PS19 mice, activation-evoked microvascular responses were markedly disrupted, leading to disordered segmental vascular adjustments and capillary RBC flux. These alterations in tissue perfusion occurred alongside a profound increase in neuronal network activity and a reduced efficiency of neurovascular coupling, resulting in diminished blood delivery to active brain regions. To assess the consequences of this mismatch between neural activity and CBF, we measured intravascular and tissue pO_2_. Baseline intravascular pO_2_ was reduced in PS19 mice, consistent with an increased OEF. During activation, OEF did not decrease as much as in WT mice, potentially inadequate tissue O_2_ delivery. Moreover, during sustained activation, tissue pO_2_ progressively declined, transiently reaching hypoxic levels in tissue farther away from arterioles. Consistent with an energetic imbalance arising from impaired O_2_ delivery^34, 68, 69^, NADH fluorescence measurements indicated a failure to re-establish metabolic homeostasis following activation. While these findings reveal the segmental hemodynamic basis of p-tau–induced neurovascular dysfunction, they also identify reduced O_2_ availability, resulting from aberrant network activity and reduced neurovascular coupling efficiency, as a previously unrecognized harmful effect of p-tau that appears well before neurofibrillary tangles, neurodegeneration, and cognitive impairment.

Studies in animal models of AD and other tauopathies have provided evidence of disrupted coupling between neuronal activity and blood flow, as well as increased capillary stalling^11, 52, 79–82^. However, the impact of these alterations on the moment-to-moment delivery of O_2_ to the brain at rest and during activation remained unclear. Here, by monitoring network activity, hemodynamic changes and intravascular and tissue pO_2_ dynamics we demonstrate that the disorganized and delayed hemodynamic response to neural activation in PS19 mice impairs microvascular O_2_ exchange with the brain tissue by increasing transit time heterogeneity and capillary stalling, ultimately compromising O_2_ delivery to the tissue. Such O_2_ exchange insufficiency, coupled with the increased O_2_ requirements driven by the aberrant network activity, resulted in reduced O_2_ availability and hypoxic transients in regions removed from arterioles. Therefore, our findings provide direct evidence that neurovascular dysfunction threatens the cerebral supply of O_2_, on which the brain’s functional integrity depends critically.

The findings of the present study highlight the key role of capillary dysfunction in the pathogenic consequence of neurovascular alterations in neurodegenerative diseases^83^. The brain depends on a constant and well-regulated deliver of O_2_ and glucose to fulfill its regionally dynamic energetic needs^21, 84^. However, functional hyperemia is thought to be associated more with the critical demands of O_2_ than glucose, which is more abundantly available to the tissue^34, 85^. O_2_ is transported from blood to brain by diffusion, and a gradient is established between blood and tissue which reflects the rate of oxygen consumption by respiring cells^36^. When the metabolic rate increases upon activation, the O_2_ gradient also increases, resulting in a drop in local tissue pO2, which is followed by rapid rebound and “overshoot” in O_2_ due to a rapid increase in the local CBF^35, 36, 86^, mediated by a highly coordinated segmental relaxation of the microvasculature^21^. At the same time, an increase in the homogeneity of capillary transit time facilitates the O_2_ exchange with the tissue^27, 51^. As shown here, when these finely regulated microvascular adjustments are disrupted in the face of increased neural network activity, the O_2_ supply of the brain is compromised. Such a significant mismatch between O_2_ supply and demand has potentially deleterious consequences for the brain’s functional and structural integrity. However, it remains to be established if in other disease models associated with neurovascular coupling dysfunction and cognitive impairment, like Aβ accumulation, ApoE4 carriage or arterial hypertension^87–89^, O_2_ delivery dynamics are also altered. Notably, since hypertension, ApoE4 carriage, and Aβ pathology are also associated with endothelial dysfunction^87–89^, it would be of interest to determine if the anticipated reduction in O_2_ availability is more severe, or if the alterations in functional hyperemia are the major driver of the limitation in O_2_ delivery to the brain.

The impact of the reduced O_2_ availability associated with dampening of functional hyperemia in the brain dysfunction and damage induced by p-tau remains to be established. The limitation in O_2_ supply especially during activation could exacerbate the deleterious neuronal effects of tau since hypoxia induces neuroinflammation, promotes oxidative stress and suppresses growth factors, thereby contributing to neurodegeneration^90^. On the other hand, reduced O_2_ availability could promote tau hyperphosphorylation and aggregation which, in turn, would enhance tau pathology. There is evidence that sustained or intermittent hypoxia increases tau-phosphorylation, effects mediated by activation of tau phosphorylating enzymes (CDK5, GSK3β etc.) or by inducing neuroinflammation^91^. Moreover, hypoxia-independent factors could also play a role. For example, tau clearance from the brain depends in large part on interstitial fluid (ISF) and cerebrospinal fluid (CSF) drainage^92–94^. Since suppression of neurovascular coupling reduces ISF/CSF clearance^95, 96^, it is conceivable that impaired functional hyperemia exacerbates tau accumulation. Thus, tau-induced neurovascular dysfunction could, in turn, promote further p-tau buildup, creating a self-reinforcing pathological cycle. These possibilities need to be tested in future experiments.

The observation that the brain’s O_2_ balance may be compromised early in the disease course in PS19 mice raises the possibility that O_2_ supplementation could be beneficial in tauopathies. O_2_-based interventions are beginning to be explored in neurodegenerative diseases^97^. Hyperbaric O_2_ treatment has been shown to reduce amyloid pathology and rescue cognitive dysfunction in animal models^98^, and to improve cognitive function and glucose metabolism in a small cohort of AD patients^99^. However, important questions remain regarding the optimal disease stage for intervention, treatment duration, O_2_ concentration and delivery modality, and the long-term sustainability of therapeutic benefits^100^. Nevertheless, the present findings provide a rationale for further investigation of O_2_ supplementation as a potential therapeutic strategy in tauopathies.

In conclusion, we provide evidence that p-tau induces a profound disruption of the coordinated, segment-specific microvascular adjustments that underlie functional hyperemia, leading to reduced red blood cell flux, increased capillary transit-time heterogeneity, and more frequent capillary stalling. These alterations, which occur at an early disease stage, impair the ability of the capillary network to satisfy the increased O_2_ demands of neuronal hyperactivity, resulting in reduced O_2_ availability and transient hypoxia at sites distant from the arteriolar supply. While the specific consequences of these alterations for tau hyperphosphorylation and aggregation remain to be elucidated, the findings unveil a novel aspect of tau pathobiology and provide support for therapies restoring the balance between O_2_ supply and demand.

## Materials and Methods

### Mice

Animal husbandry and the experimental procedures used in this study were approved by the Institutional Animal Care and Use Committee (IACUC) of Weill Cornell Medicine (protocol number 0711–687 A) and comply with the ARRIVE guidelines^101^. Studies were performed in 3 months old mice of both sexes. Both NOS1cre (#017526)^102^ and PS19 (#008169)^46^ breeders with C57BL/6J genetic background were purchased from the Jackson Laboratory and bred in house. Sample size estimations were performed using G*Power software, guided by effect sizes reported in prior literature where available. Experimental protocols were designed to ensure adequate statistical power to detect moderate and biologically relevant differences.

### Animal preparation for *in vivo* imaging

Optical access to the brain was achieved via a chronically implanted, glass-covered cranial window, as described previously^47^. Briefly, dexamethasone (0.2 mg/kg, subcutaneous) was administered ~2 hours before surgery to reduce brain swelling. Mice were anesthetized with isoflurane (1.5–2% in room air) and maintained at 37 °C using a feedback-controlled heating blanket (SomnoSuite with RightTemp, Kent Scientific). Following removal of the scalp and periosteum, a custom titanium headpost was affixed to the right hemisphere over the somatosensory cortex using dental cement (C&B Metabond, Parkell). A bilateral craniotomy (3-mm diameter) was made over the parietal cortex using a dental drill. For all neuronal GCaMP labeling experiments, 1 μL of AAV9-Synapsin-GCaMP7f virus (Addgene, 104488-AAV9, titer: 3 × 10^13 GC/ml) was injected directly into the brain at a rate of 100–150 nL/min using a Nanoliter injector (WPI, 504127). The exposed brain was covered with sterile saline and sealed with a 3-mm square borosilicate glass coverslip (CS-3S, Warner Instruments) using cyanoacrylate glue and tissue adhesive (1469SB, 3M). For tissue oxygen recordings, a small craniotomy was made posterior to the main cranial window, near the midline and anterior to lambda. A chronic cannula (C315GS-5/SP and C315DCS-5/SPC; Protech International, Inc.) was implanted into this site toward cranial window and securely affixed to the titanium headpost using additional dental cement. For experiments requiring targeted expression of GCaMP in Nos1-expressing neurons, AAV-PHP.eB-Syn-FLEX-GCaMP7f (Addgene #104492-PHPeB) was delivered after surgery via retro-orbital injection, diluted in sterile saline to a final concentration of 1 × 10^11^ GC per 100 μL. After the 7-day recovery period, mice then underwent a two-week habituation period to head-fixation and air-puff, after which awake imaging data were collected. In a subset of mice, the femoral artery was cannulated under isoflurane anesthesia to monitor arterial pressure and blood gases during head restraint. These mice maintained stable physiological parameters (extended data Fig. 1a), indicating minimal stress.

### Two-photon imaging of the whisker barrel cortex in awake mice

To locate the barrel cortex for awake imaging, laser speckle contrast imaging (Omegazone, Omegawave) was first performed under light anesthesia (0.2% isoflurane) as described previously^11^. The resulting functional maps were used to localize the whisker barrel field. Subsequently, two-photon microscopy under anesthesia was used to acquire low-magnification (5×) cortical maps and high-resolution (25×) vascular image stacks for reference. Functional and structural maps were acquired days before awake imaging to avoid any residual influence of isoflurane anesthesia.

For awake imaging, mice were briefly anesthetized (1.5–2% isoflurane for ~1 min) and injected intravascular tracers to label the vasculature. Mice were head-fixed on a saucer platform under the two-photon microscope and received sweetened milk rewards during imaging. Whisker stimulation was delivered via a Picospritzer (~10 psi) as continuous 5 s or 30 s air-puff trains to the contralateral whiskers, generating gentle flickering without distress. Stimulation was synchronized with imaging using a TTL pulse generator (OTPG_4, Doric Lenses) triggered from the Olympus breakout box, with parameters controlled in Doric Studio. Simultaneous behavioral monitoring was performed to identify periods of movement during image acquisition. Mouse behavior was recorded at 120 fps using an infrared webcam. An Arduino microcontroller triggered the video recording in sync with two-photon acquisition, enabling precise alignment between motion events and imaging data.

### Two-photon imaging of the microvasculature and GCaMP signals

Imaging was performed on a Fluoview 2-photon microscope (FVMPE; Olympus) equipped with a 25 × 1.05 NA water-immersion objective (Olympus), with excitation pulses delivered by a femtosecond laser (InSight DS+; Spectra-Physics). For vessel labeling, we used either FITC- or Texas Red–conjugated dextran injected retro-orbitally (i.v. 50 μL, 2.5% w/v, Invitrogen) and set the excitation wavelength to 900 nm. For simultaneous calcium imaging and vascular labeling, GCaMP7f and Texas Red were excited at 910 nm. The laser beam was directed through a primary dichroic mirror (FV30-NDM690), followed by an IR blocking filter (FV30-BA685RXD), and guided to gallium arsenide phosphide (GaAsP) photomultiplier tubes (PMTs) via the FV30-SDM-M mirror. Fluorescence signals were collected using an FV30-FGR filter cube (green: 495–540 nm; red: 575–645 nm, separated by a 570 nm dichroic mirror) mounted in front of the GaAsP PMTs. Image stacks and movies were acquired using Fluoview software (FV31S-SW, version 2.3.1.163; Olympus). For microvascular segment analysis during functional hyperemia (Fig. 1), the objective was mounted on a piezo stage (P-725K085, Physik Instrumente) for rapid volumetric imaging. Arterioles and venules were identified based on blood flow direction and vessel structure^11, 47, 50^. Fast image stacks were acquired using resonant scanner at a rate of less than one second per volume, capturing at least three distinct segments of arteriolar structures from the pial surface into deeper cortical layers, following the vascular architecture. The field of view was zoomed in to focus on the targeted structures, and each stack was acquired at a resolution of 512 × 512 pixels. For line scans, we used galvanometer scanners to repeatedly scan a parallel short line centered along the inner lumen of the target vessel, with a line acquisition rate of 0.8–1.3 kHz for at least 30 seconds. For capillary and microvascular geometry analyses, we acquired image stacks with 1 μm z-steps using galvanometer scanners, with a frame rate of 1.087 Hz and spatial resolution of 1.0057 pixels/μm. For imaging GCaMP7f and intravenously injected Texas Red dextran, two-channel movies were acquired using a resonant scanner at 3.8 Hz with 4-line averaging (1.0057 pixels/μm) over a 5-minute recording duration.

### Analysis of vessel diameter

For vessels oriented parallel to the imaging plane and imaged at a single time point, we generated maximum intensity z-projections of the stacks in ImageJ and calculated vessel diameter using a full-width at half-maximum (FWHM) approach. For time-lapse 4D recordings, fast image stacks were first processed in ImageJ to generate maximum intensity z-projections at each time point. The resulting stack was then analyzed using custom MATLAB code that extracts vessel diameters based on Radon projections and FWHM calculations^103^. The diameter change over time was detected for each vascular segment. Values from each vessel type were averaged per mouse for statistical analysis. For vessels oriented perpendicular to the imaging plane (penetrating arterioles), diameters were measured using the Thresholding in Radon Space (TiRS) method (https://www.drew-lab.org/code)^104^. Arteriolar diameter time series were processed with a second-order zero-phase low-pass filter (MATLAB functions: butter, filtfilt) before further analysis. Trials involving 5 seconds air puff stimulation, vascular responses were assessed by the peak dilation observed within the stimulation period. In the 30 second stimulation paradigm, two components were measured: the first peak, representing the maximum dilation between 1–4 seconds after onset, and the shallow peak, calculated as the average diameter during the sustained phase between 20–30 seconds of stimulation. For all protocols, percent change in diameter was determined by comparing the peak or shallow-peak diameter to a baseline, which was obtained by averaging the vessel diameter over the 5 seconds immediately preceding stimulation. Latency was measured as the time required to reach half of the maximum dilation.

### RBC speed and flux

Space–time images from line scans displayed diagonal streaks, whose slopes were inversely related to centerline RBC speed. All scans were processed in MATLAB using a Radon transform–based algorithm to compute line integrals of the 2D image g(x,y) at multiple angles (https://github.com/sn-lab/Blood-flow)^105, 106^, enabling estimation of RBC speed over time. The baseline RBC speed was calculated as the average over the 5 seconds immediately preceding stimulation, and the speed increase was defined as the maximum RBC speed observed during stimulation. Capillary RBC flux was measured from line scans on the venular side of the capillary, which were converted into 500-line image stacks. RBC events—identified as valleys in the binary-segmented intensity profile—were manually counted in every other frame. For each frame, flux was calculated as RBC count ÷ acquisition time for the 500 lines (RBCs·s^−1^). The mean flux for each capillary segment was obtained by averaging across the sampled frames. Flux variability was quantified as the standard deviation (STD) and coefficients of variation (CV), with CV defined as the ratio of STD to the mean flux.

### Capillary stall counts

Methods to assess capillary stalling in our lab have been published^52, 107^. Briefly, first we counted the number of capillary segments in each image stack. Stacks were duplicated and cropped to remove the top 40–120 μm containing the meningeal layers and large pial vessels. Vascular skeletons were generated in ImageJ using the TubeAnalyst macro (IRB Barcelona), which detects and returns counted vascular segments on the skeleton. These skeletons were merged with the original image stacks, and missing segments were manually corrected, particularly near the pial surface and stack edges where imaging artifacts disrupted automated detection. Capillary stalls were identified by visually tracking RBC movement; because fluorescent dextran labels plasma but not RBCs, the cells appeared as dark patches or streaks within the lumen, with their consistent presence indicating blood flow. Capillary stalls were calculated as the proportion of stalled capillary segments relative to the total number of imaged capillary segments^52^. For each stall, upstream and downstream branches were examined to determine proximity to PA or AV. Most were located near AV so branching order relative to the AV was then quantified.

### Analysis of GCaMP signals

GCaMP7f analysis was done as described^47^ previously. Briefly, GCaMP7f movies were motion-corrected using the NoRMcorre algorithm in EZcalcium^108^. nNOS neuron were manually outlined in CalciumDX^109^, and fluorescence time series (ΔF/F) were extracted with custom MATLAB scripts. Baseline fluorescence was calculated as the mean of the lowest 25% of values in a 15 s sliding window for 5 s air-puffs. Signals were low-pass filtered (4th-order, zero-phase) and considered active when ΔF/F exceeded 2 SD above baseline, with termination defined as <0.5 SD above baseline. Activation time accounted for signal rise, defined when the ΔF/F derivative exceeded 0.5 SD. Ensemble activity was calculated as the percentage of active cells per frame, with significant ensemble events identified when activity exceeded chance levels^57^. Specifically, the threshold was estimated by randomly lag-shuffling each neuron’s activity relative to others, computing the number of simultaneously active cells at each time point and repeating this process 1,000 times to derive a 99.9% confidence interval. Analyses were restricted to datasets with minimal motion (~10% of total recording time), as calcium activity in awake mice is strongly influenced by locomotion. Statistical analyses included all imaging sessions from multiple animals per genotype. When more than one session was obtained from the same mouse, recordings were collected from non-overlapping fields of view, ensuring distinct cell populations.

### O_2_ imaging using Oxyphor 2P imaging

For intravascular O_2_ measurements, mice were briefly anesthetized with isoflurane (1.5–2%) for ~1 minute, followed by retro-orbital injection of Oxyphor 2P (~34 μM, 0.1 ml) and FITC-dextran to label blood vessels (50 μL, 2.5% w/v). For tissue (extravascular) O_2_ measurements, 2 μl of 125 μM Oxyphor 2P was infused through a chronically implanted cannula (see Animal Preparation for in vivo imaging) at 0.1 μl/min at least 2 hours before imaging, along with intravenous FITC-dextran (100 μL, 2.5% w/v) for vascular labeling. Fluorescence and two-photon phosphorescence lifetime imaging (2PLM)^110^ was performed on our commercial Olympus Fluoview scope modified for 2PLM operation. Each phosphorescence excitation/collection cycle consisted of a 10 μs-long train of femtosecond pulses (80 MHz repetition rate), followed by a 290 μs collection period for phosphorescence photons. Galvo-scanner movement between consecutive locations took 1.7 ms and was included in the imaging time. To optimize spatial coverage, we sequentially acquired 100 phosphorescence cycles at each of 7 ROIs, repeated this sequence 140 times, and then averaged each pair of repetitions. This resulted in a final temporal resolution of 70 data points over the course of 1 minute, consisting of 15 seconds of baseline, 30 seconds of whisker stimulation and 15 seconds of recovery. For tissue oxygen measurements at a higher spatial resolution, a 32 × 32 grid (1,024 ROIs) was applied across the imaging area. At each ROI, 200 phosphorescence cycles were acquired, and the entire sequence was repeated 5 times (which took 5 minutes 12 seconds). Periods of mouse movement were excluded from the analysis. Excitation laser was set at 960 nm and emitted light passed through a custom ordered primary dichroic mirror (NDM 860, Olympus) and an IR blocking filter (BA850, Olympus) and was directed to a red-sensitive GaAs photomultiplier tube (H7422P-50, Hamamatsu) via the FV30-SDM-M mirror through a bandpass filter (755/35 nm, Semrock). The PMT signal was digitized using a 1 MHz analog-to-digital converter and routed to the Olympus breakout box for visualization in Fluoview. In some datasets, Olympus’s built-in multialkali detectors was used. Microscope control for lifetime imaging was managed through the Multi-Point Mapping Advanced Software (MMASW) module in Fluoview. During experiments, a custom-made electric heater warmed the objective lens to maintain the temperature of the immersion medium between the cranial window and the objective at 36–37 °C. The immersion medium was also pre-heated to 37 °C.

### O_2_ data analysis

Two-photon lifetime imaging data from the Olympus system were acquired in the format of ROIs (rows) by time points (columns). Image stacks were opened in ImageJ, and the green and far-red channels were separated; both channels were saved as tiff file for subsequent analysis. Data were processed using custom MATLAB scripts to average phosphorescence intensity decays for each ROI, and to temporally average traces over defined time bins. The data were fit to single-exponentials using nonlinear least-squares methods to obtain fluorescence lifetimes, which were converted to pO_2_ values using a set of parameters obtained in independent experiments, as previously described^62^. The dextran channel was used to verify that excitation occurred at the correct spatial location—particularly important for measurements in awake animals, which can move resulting in distorted measurements. Hemoglobin oxygen saturation (sO_2_) was derived from pO_2_ measurements using the Hill equation with parameters specific to C57BL/6 mice (h=2.59, P_50_ = 40.2 mmHg^111^ where h is the Hill coefficient and P_50_ is the pO_2_ at 50% hemoglobin saturation^64^. Oxygen extraction fraction (OEF) was computed for each set of measurements as: (SO_2,A_–SO_2,V_)/SO_2,A_, SO_2,A_ and SO_2,V_ denote SO_2_ in arterioles and venules, respectively^51^.

The validity of applying the calibration coefficients for lifetime-pO_2_ conversion, obtained using a different system^62^, in our current two-photon microscopy setup was verified using an additional reference system. A sealed POC-R2 perfusion chamber (Pecon) was mounted on the stage of a Leica microscope, equipped with a temperature controller, and connected to a perfusion system. The chamber was filled with a solution containing 125 mM KCl, 0.2 mM EGTA, 20 mM HEPES-Tris, 1 mg/ml BSA, 10 mM cytochrome c and 0.5 μM Oxyphor 2P. Mouse brain mitochondria (0.2 mg/ml) and succinate (10 mM) were added to the chamber, after which perfusion was stopped. The respiration of the mitochondria resulted in a continuous decline in oxygen concentration in the chamber, which, for the conditions described, occurred at a well-defined and reproducible rate^112^. Thus, at any given time point, the concentration of oxygen in the chamber could be determined based on that rate, providing the reference data for an independent phosphorescence lifetime/pO_2_ calibration plot. As expected, the calibrations obtained using this method and the method described previously^62^ were found to be very close (extended data Fig. 3f)

### Two-photon imaging and analysis of NADH autofluorescence

Mice were injected retro-orbitally with 100 μl of 5 mM SR101 (Sigma-Aldrich, S7635; freshly prepared from powder on the day of injection) 4 h before imaging for astrocyte/oligodendrocyte labeling and hemodynamic artifact correction in NADH signals^113^. Two-photon excitation was performed at 740 nm, and emission was collected simultaneously in the blue (410–460 nm, NADH autofluorescence) and red channels (495–540 nm, SR101-labeled cells) using GaAsP detectors. Two channel movies were acquired using galvanometer scanners at a frame rate of 1.087 Hz and spatial resolution of 1.0057 pixels/μm over 2 minutes. Each recording began which 15 seconds of baseline followed by 30 seconds of whisker stimulation. Images were imported into imageJ, and regions of interest were drawn to avoid shadows from large vessels^70^. SR101 signals were then used to correct the NADH signals for hemodynamic optical effects, following a previously reported method^68^.

### Assessment of mitochondrial function *ex vivo*

Mitochondrial oxygen consumption, the release of H_2_O_2_, and membrane potential were measured using a high-resolution respirometer (O2K oxygraphy, Oroboros Instruments, Innsbruck, Austria) equipped with two-channel optical setup to monitor fluorescence (excitation/emission 525/580 nm)^112^ at 37°C. Mitochondria isolated from the neocortex were added to the oxygraph chamber to a final concentration of 0.05 mg/ml of protein. Chamber contained 2 ml measuring buffer composed of 125 mM KCl, 0.2 mM EGTA, 20 mM HEPES-Tris, 4 mM KH2PO4, pH 7.4, 2 mM MgCl2, 1mg/ml BSA, and supplemented with specific substrates. The following substrates were used: 2 mM malate and 5 mM pyruvate (for complex-I supported respiration), or 5 mM succinate and 1 mM glutamate (for complex II). After recording oxygen consumption rate in non-phosphorylating conditions (state 2), 200–400 μM ADP was added to initiate state 3 (phosphorylating) respiration. If H_2_O_2_ release was simultaneously assessed in the same assay, the measuring buffer was supplemented with 10 μM Amplex UltraRed (Invitrogen), 4 U/ml horseradish peroxidase, and 5 U/ml superoxide dismutase. The raw resorufin fluorescence was calibrated by the addition of H_2_O_2_ aliquots of known concentration (250 nM) from a fresh standard solution of H_2_O_2_ (ε_240nm_=46.3 mM^−1^cm^−1^). If membrane potential was assessed, 1 μM safranine O was added instead of H_2_O_2_ detection system, followed by the addition of 100 nmol ADP; fluorescence was measured as described in Ref^114^. Respiration rates and fluorescence emission data were recorded using the DataLab software (version 6.1.0.7) at 1 Hz time resolution and analyzed in Microcal Origin. Oxygen consumption in nmol O_2_/min or H_2_O_2_ release in nmol H_2_O_2_/min was normalized by the amount of protein in mg added to the buffer. All results are expressed as means±SD for at least three biological replicates.

### Mitochondrial complex quantification by flavin fluorescence

Sample solubilization with DDM and running conditions for high-resolution clear native (hrCN) electrophoresis of mitochondrial membranes were performed as previously described^115^ to determine the absolute content of mitochondrial complex I and α-ketoglutarate dehydrogenase. Gels were scanned for flavin fluorescence in a Typhoon 9000 gel scanner (GE) using a 473 nm laser and BPB1 filter (530 nm maximum, 20 nm bandpass). Flavin signal calibration was performed using standard solutions of FMN.

### Neuronal recording in brain slices

Procedures for whole-cell patch clamp recordings in brain slices from our laboratory have been published^89, 116^. Briefly, mice were anesthetized with 2% isoflurane, and brains were rapidly removed into ice-cold sucrose based ACSF (s-ACSF; in mM: 248 sucrose, 26 NaHCO_3_, 1 NaH_2_PO_4_, 5 KCl, 5 MgSO_4_, 0.5 CaCl_2_, 10 glucose; 95% O_2_ / 5% CO_2_; pH 7.3). Coronal neocortical slices (200 μm) were prepared using a Leica VT1000s vibratome (Leica Microsystems) and incubated for 1h at 32°C in lactic acid-ACSF (in mM: 124 NaCl, 26 NaHCO_3_, 5 KCl, 1 NaH_2_PO_4_, 2 MgSO_4_, 2 CaCl_2_, 10 glucose, 4.5 lactic acid; 95% O_2_ / 5% CO_2_; pH 7.4). After a 1-h recovery, slices were transferred to a glass-bottom recording chamber (P-27; Warner Instrument) on a Nikon E600 epifluorescence microscope and perfused with oxygenated l-ACSF (1 ml/min, 30–32 °C). Layer 2/3 pyramidal neurons were identified by their characteristic soma morphology and targeted for whole-cell patch-clamp recordings. Patch electrodes were pulled from borosilicate capillaries (OD 1.5 mm/ID 0.86 mm; World Precision Instruments) using a P-80 micropipette puller (Sutter Instruments) and had resistance of 4–5 MΩ when filled with intracellular solution containing (in mM: 130 K-gluconate, 10 NaCl, 1.6 MgCl_2_, 0.1 EGTA, 10 HEPES, and 2 Mg-ATP, adjusted to pH 7.3). Whole-cell current-clamp recordings were obtained using a Multiclamp 700B amplifier (Molecular Devices) with signals filtered at 2 kHz and digitized at 10 kHz (Axon Digidata 1550B, Molecular Devices). After forming a giga-Ω seal, brief negative pressure was applied to achieve whole-cell configuration. Access resistance and membrane parameters were monitored continuously, and only cells with stable access resistance (change <10%) were included. Action potentials spikes were recorded in current-clamp mode, and action potentials were evoked by +35 pA current injection. Membrane potential and spike frequency were analyzed offline using pClamp 11 software (Molecular Devices). Liquid junction potentials were corrected during offline analysis.

### Data and statistical analysis

All 2-photon microscopy images were preprocessed with ImageJ (NIH) analyzed using custom written code on Matlab (R2018a, R2021b, R2022a, R2023b, R2024b Mathworks). Statistical analysis was performed using GraphPad Prism 10 (GraphPad Software, Inc). Animals were randomly assigned to experimental groups. Animals exhibiting poor cranial window quality—characterized by excessive inflammation or obscured structural visibility—were excluded from imaging experiments. Statistical analyses were performed using nested t-tests or two-way ANOVA / mixed effect analysis (factors: time, vessel segment, or genotype) followed by Tukey’s post hoc correction or Fisher’s LSD where appropriate. For datasets with a single value per animal, group comparisons were performed using unpaired t-tests for normally distributed data with equal variances, Welch’s t-tests when variances differed, and Mann–Whitney U tests when normality assumptions were unmet. All statistical tests were two tailed. Differences in regression slopes between WT and PS19 mice were evaluated using ANCOVA in MATLAB, incorporating an interaction term (PctChange ~ Ensemble × Genotype). The significance of the Genotype × Ensemble interaction tested whether slopes differed between groups.

## Extended Data

**Extended Data Figure 2:**
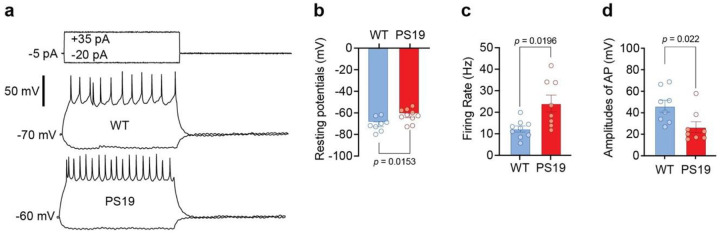
Patch clamp recordings of pyramidal neurons in WT and PS19 mice **a.** Representative traces in whole-cell current-clamp mode showing decreased resting membrane potential and increased spiking activity induced by a depolarizing pulse in PS19 compared to WT mice. **b.** Resting membrane potential was more depolarized in PS19 neurons than in WT neurons, indicating greater excitability; unpaired t-test. **c.** Increased firing rate in PS19 neurons, consistent with neuronal hyperactivity; Welch’s t-test. **d.** Reduced action potential amplitude in PS19 is consistent with increased firing rates and membrane depolarization; unpaired t-test. All data are from 6 WT and 7 PS19 mice.

**Extended Data Figure 3:**
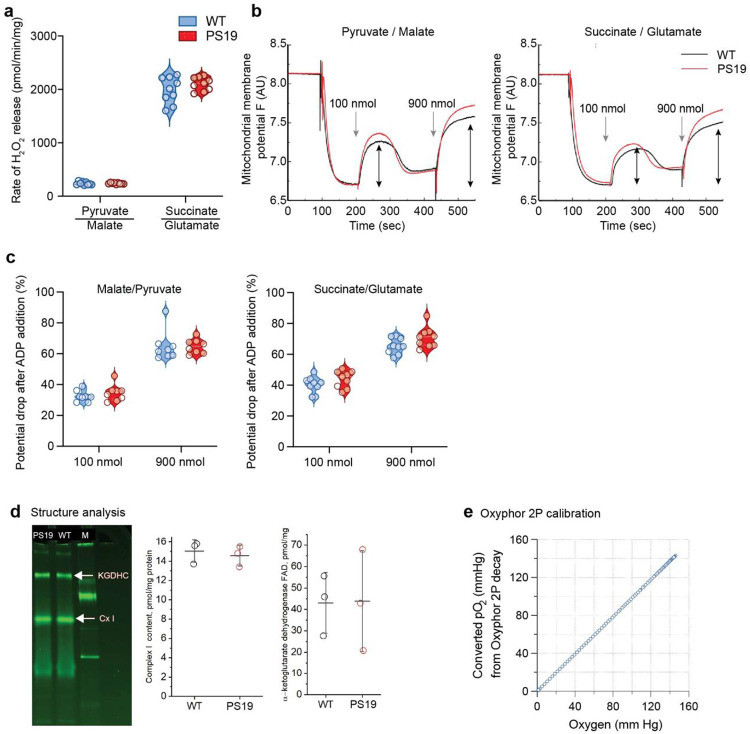
Mitochondrial function and content analysis in WT and PS19 mice **a.** The reactive oxygen species output under each substrate condition did not differ between WT and PS19. **b.** The ability of neocortical mitochondria to maintain the proton gradient required for ATP synthesis was maintained in both WT and PS19. **c.** ADP-stimulated respiration and ATP synthase activity were similar between WT and PS19 mice. **d.** Mitochondrial protein content analysis showing comparable levels of complex I and alpha-ketoglutarate dehydrogenase (FAD) between WT and PS19 mice. **e.** Calibration curve relating chamber oxygen levels to Oxyphor 2P phosphorescence lifetime measurements used for in vivo pO_2_ quantification. In panel **a-d** data are shown as median and interquartile range; 3 mice per genotype (3–4 technical replicates/mouse); statistical comparisons were performed using a mixed effects model.

## Data Availability

The authors declare that the data supporting the findings of this study are available within the article and Supplementary Information files.
